# The role of parietal cortex in the formation of color and motion based concepts

**DOI:** 10.3389/fnhum.2014.00535

**Published:** 2014-07-28

**Authors:** Samuel W. Cheadle, Semir Zeki

**Affiliations:** Wellcome Laboratory of Neurobiology, University College LondonLondon, UK

**Keywords:** concept formation, parietal cortex, category learning, V4, V5

## Abstract

Imaging evidence shows that separate subdivisions of parietal cortex, in and around the intraparietal sulcus (IPS), are engaged when stimuli are grouped according to color and to motion (Zeki and Stutters, 2013). Since grouping is an essential step in the formation of concepts, we wanted to learn whether parietal cortex is also engaged in the formation of concepts according to these two attributes. Using functional magnetic resonance imaging (fMRI), and choosing the recognition of concept-based color or motion stimuli as our paradigm, we found that there was strong concept-related activity in and around the IPS, a region whose homolog in the macaque monkey is known to receive direct but segregated anatomical inputs from V4 and V5. Parietal activity related to color concepts was juxtaposed but did not overlap with activity related to motion concepts, thus emphasizing the continuation of the segregation of color and motion into the conceptual system. Concurrent retinotopic mapping experiments showed that within the parietal cortex, concept-related activity increases within later stage IPS areas.

## Introduction

Abstraction is a key part of concept formation, involving the ability to generalize across particular instances, to generate knowledge of abstract categories and subsequently concepts (Medin and Smith, 1984). It is linked to segmentation and grouping through a shared reliance on relational knowledge that binds instances, thereby abstracting from the particular to the general. The parietal cortex is known to be involved in grouping and segmentation (Zeki and Stutters, 2013), leading to the hypothesis that it may also play a key role in generalizing beyond stimulus properties. In the work reported here, we examine the role of the parietal cortex in the formation of categorical and conceptual knowledge, and whether the principle of functional specialization (Zeki et al., 1991), which seems to apply for the grouping and segmentation of stimuli according to color and motion in parietal cortex, also applies to the formation of concepts related to the two attributes. This seemed plausible, given that grouping is a step in the formation of concepts.

In theory, it is possible that the same cortical area(s) could be engaged in grouping and the formation of concepts, since both are abstract processes that can be applied to an almost infinite variety of stimuli and conditions. In practice, however, the grouping of stimuli according to color or to motion engage distinct sub-divisions of parietal cortex (Zeki and Stutters, 2013), thus raising the question of whether the formation of concepts based on these two attributes also engages distinct networks within the same areas, and especially within parietal cortex. A body of work has highlighted the role of the parietal cortex in category learning and concept formation. Using electrophysiological recordings (Freedman and Assad, 2006) have demonstrated that newly learned motion based categories are robustly reflected in the firing of cells within the lateral intraparietal (LIP) area, whose activity reflects learnt motion categories, rather than specific directions of motion (as is the case for V5 neurons, Zeki, 1978; Zeki et al., 1991). Importantly, categorical selectivity within the LIP is not limited to cases in which the categories are defined by motion signals, being also present for shape-defined categories (Fitzgerald et al., 2011). It has therefore been proposed that processing within this parietal region reflects an important transformation from the “raw” perceptual representation into a meaningful and behaviorally relevant signal, crucial for category and concept formation (Fitzgerald et al., 2011; Freedman and Assad, 2011); an idea that is reinforced by the demonstration that grouping of stimuli according to color or motion engages the parietal cortex. Our principal aim in this study therefore was to learn whether human parietal cortex plays a role in the formation of concepts based on color and motion and, if so, whether it is the same or contiguous regions of parietal cortex that are involved. If the principles of functional segregation are maintained, non-overlapping and separable areas in parietal cortex should be engaged in the formation of concepts based on these two attributes.

Parietal cortex receives inputs from earlier visual areas, thus opening up the possibility that areas such as V4 and V5, traditionally considered as being perceptual in function, may also contribute to concept formation related to their attributes. Thus, a secondary aim of this work was to learn whether V4 and V5 are also engaged in concept learning. We chose to employ color and motion based concept stimuli similar to those of two recent studies (Freedman and Assad, 2006; Mirabella et al., 2007) because color and motion are readily separable psychophysically and perceptually (Ramachandran and Gregory, 1978; Cavanagh et al., 1984; Moutoussis and Zeki, 1997; Cheadle and Zeki, 2011), and the areas most responsive to these features (V4 and V5) are well delimited anatomically (Zeki et al., 1991).

**Figure 1:**
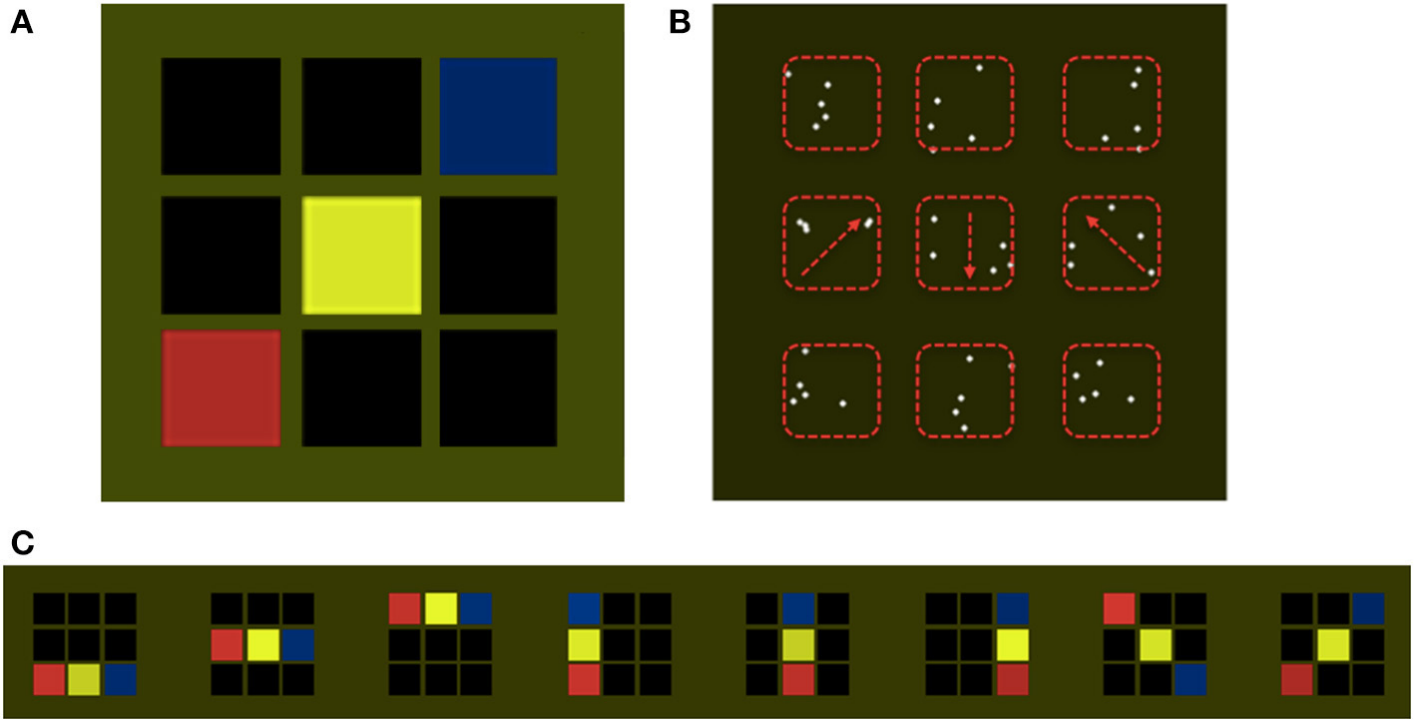
**Stimulus examples**. Participants were trained to recognize patterns belonging to a target concept using visual displays consisting of specific sequences of color or directions of motion. Panel **(A)** shows an example of a target concept sequence, consisting of three colored squares embedded in a 3 × 3 grid. This example follows the concept rule, red precedes yellow precedes blue, from left to right (but in the actual experiment could also be bottom up, as shown in panel **(C)**. Panel **(B)** displays an equivalent example for the motion condition in which, each square is replaced by a set of white dots. Three sets of dots were moving (marked by the red arrows, not shown to observers), and all other sets of dots were stationary. Red dashed rectangles (not shown to observers) mark the boundaries of the areas in which dots could be displayed. The concept consisted of sequences of moving dots, with directions 45°, 180°, or 315°. Panel **(C)** displays all patterns belonging to the target color concept, including the example displayed in Panel **(A)** (far right). For all of the patterns displayed the relational properties between elements remain constant, regardless of the spatial position of the colored squares, i.e., Red precedes yellow precedes blue, from left to right, or from bottom to top. Diagonal patterns follow the left to right rule. This constant relationship between the colors or the directions of motion constitutes the invariant feature defining the target concept.

Our study consisted of a training phase and a test phase. During the training phase participants learned to recognize items belonging to a novel concept based on color or motion associations (Figure 1), and distinguish them from other color or motion stimuli to which no concept was attached. During the test phase participants used the newly acquired concepts to perform a delayed-match-to-concept (DMC) task (Freedman and Assad, 2006, Figure 2). We hypothesized that separate regions of the parietal cortex, in and around the intraparietal sulcus, would be engaged during the formation of concepts based on color and motion.

**Figure 2:**
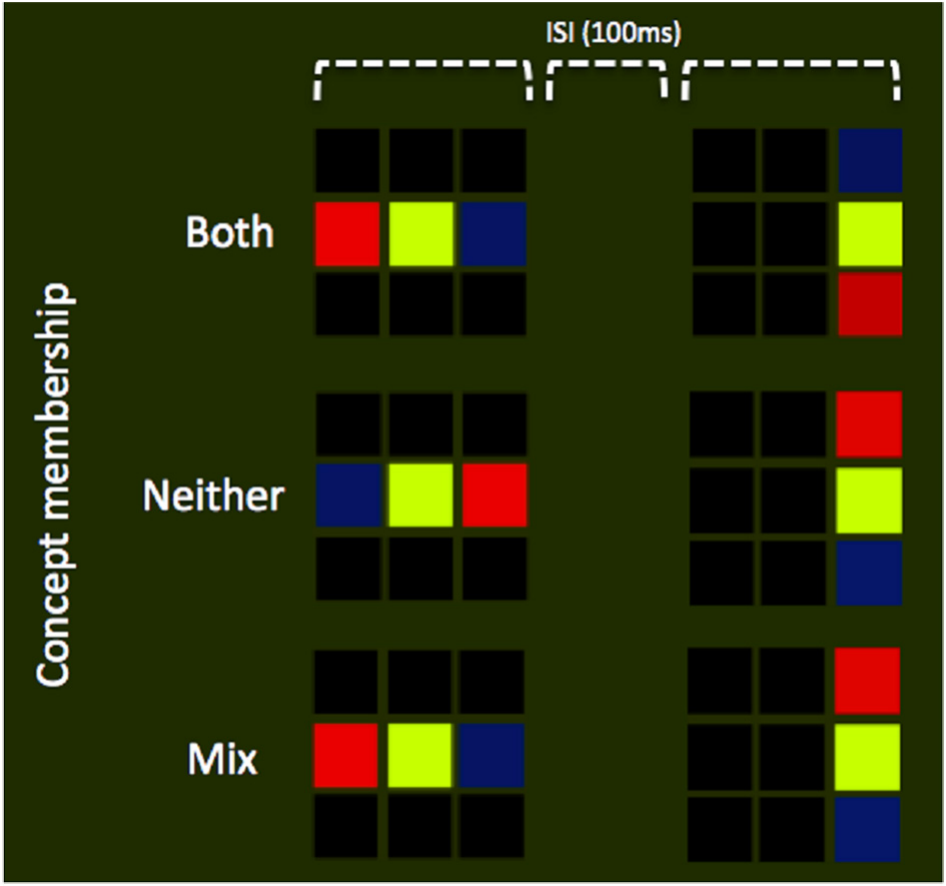
**Experimental design**. Schematic illustration of display sequences, in which all trials consisted of two stimulus presentations each with a duration of 1 s. Separating these two presentations was a blank screen, ISI interval of 100 ms. Participants were instructed to respond on the basis of the number of patterns that matched the target concept. Three possible combinations are illustrated in the panel above: (1) Both stimuli are drawn form the target concept set (top row; see Figure 1, for the rules on concept membership), (2) Neither stimuli are drawn from the target concept set (middle row), or (3) a single stimulus is drawn form the target concept set (bottom row).

## Methods

### Participants

Sixteen participants ranging in age from 20 to 40, all with normal or corrected to normal vision, took part in the study (approved by the UCL Ethics Committee); written consent was acquired from all, in accordance with the Declaration of Helsinki (World Medical Organization, 1964/1996).

### Stimuli

The target concept was defined as relational knowledge linking spatially distributed heterogeneous visual patterns, according to specific (and artificially generated) rules. The target concept consisted of sequences of features (color or motion) having a fixed relationship. For the color condition each pattern was composed of three colored squares (red, yellow, or blue), presented inside a 3 × 3 grid of black squares (Figure 1A). The color–based concept was that the sequence of colors should consist of red-yellow-blue from left to right or bottom to top (Figure 1C). Each square subtended 4° × 4° of visual angle, with the entire 3 × 3 grid filling a 15° × 15° area. For the motion condition each sequence was composed of three sets of moving dots, with motion directions of 45°, 180°, or 315°, embedded within a 3 × 3 grid containing stationary sets of dots (Figure 1B). Each set was composed of 5 dots (radius = 0.1°) and all dots moved at 7 s^−1^ within a rectangular area of 4° × 4°. The motion-based concept was motion in the 2, 6, and 10 o'clock directions (that is, up and to the right, downwards, and up and to the left) when presented left to right, and the complementary set of directions, rotated 90° anti clockwise, when presented bottom to top. Concept sequences were fixed across participants. Non-concept items, either in the color or motion domain, were randomly permuted sequences that did not conform to the concept rules, i.e., the sequence, blue, yellow, red (left to right), constituted a non-concept item in the color domain, and the sequence of directions 6, 2, and 10 o'clock (left to right), constituted a non-concept item in the motion domain. Stimuli were created and displayed using Matlab and Cogent Graphics (developed by John Romaya at the LON at the Wellcome Department of Imaging Neuroscience).

### Training

Participants were first trained outside the scanner on the concept recognition task based on color or motion, using the stimuli described above. First, they were presented with stimulus examples, and informed which were concept members. This was reinforced through a training task in which the subject was required to judge the concept membership of individual stimuli (i.e., a single pattern of 3 × 3 pattern). Each stimulus was presented until a response was made, after which feedback (a beep for incorrect responses) was given. Subjects completed four blocks (two color, two motion) of this task. Finally, to ensure that they had learned to discriminate between concept and non-concept stimuli successfully (above 80% performance), they were tested on the main version of the experiment, a delayed-match-to-concept (DMC) task, which was also used in the scanning session (Figure 2). In this version, trials consisted of two stimulus presentations each with a duration of 1 s. Separating these two presentations was a blank screen inter-stimulus-interval (ISI) of 100 ms. There was an inter-trial-interval (ITI) of between 1.65 and 2.15 s, depending on a random jitter varying between 0 and 500 ms; this resulted in a total trial duration between 3.75 and 4.25 s. Participants were instructed to respond at the end of each trial, based on the number of patterns that matched the target concept. The three possible stimulus combinations were (a) both concept members, (b) neither a concept member, and (c) a mixture, i.e., the first OR the second item was a concept member. The latter was considered a condition of no interest and is not reported in the results. Participants were instructed to press button “A” for a *mixture* and button “B” when both items were of the same type (i.e., either *both* or *neither* were concept members). This design aimed to equalize attentional requirements across conditions and, through the dual stimulus presentation, generate a sustained concept-related BOLD response. All training sessions took place no more than 2 days before the scanning session.

### Testing

The study used an event related design in which the stimuli either belonged, or did not belong, to a learned concept, using the DMC task described above. Each subject took part in four runs (two color/two motion), each consisting of 120 trials, in which the presentation order of conditions was pseudo-randomized. Of these 120 trials per run, 40 were from the concept condition, 40 were from the non-concept condition, and 20 from the mixed condition. Null trials (no stimulus present, and no task performed) were also included, comprising the remaining 20 trials.

### Image acquisition

MRIs were acquired using a 3-T scanner equipped with a standard transmit–receive head coil (Magnetom Allegra, Siemens Medical). An echo planar imaging (EPI) sequence was applied for functional scans, measuring BOLD signals (*TR* = 2.4 s, *TE* = 30 ms, matrix size = 64 × 72, slice thickness = 2 mm, gap between slices = 1 mm, field of view = 192 × 192 mm^2^). Each brain image was acquired in a descending sequence comprising 40 oblique axial slices covering the whole cerebral cortex. Four runs were performed within each scanning session, each comprising 210 volumes. Anatomical images were acquired in the sagittal plane to obtain a high-resolution structural image (176 slices per volume; isotropic resolution = 1 × 1 × 1 mm, *TR* = 7.92 ms, *TE* = 2.4 ms). Field maps were also acquired with the Siemens standard gradient-echo field map sequence for correcting geometric distortion of EPI images (Hutton et al., 2002).

### Preprocessing and analysis

SPM-8 was used to preprocess the data following the steps of realignment, coregistration, normalization, and smoothing. Head movement parameters were calculated from the realignment output and included as regressors of no interest in the general linear model (GLM). For each subject, the stimulus functions (boxcars based on onsets and durations) were convolved with the default SPM8 canonical haemodynamic response function and entered into a linear convolution model. The following contrasts were generated (revealing voxels that display greater BOLD response): (1) *task* > *baseline*, (2) *color* > *motion*, (3) *motion* > *color*, (4) *color concept* > *non-concept*, and (5) *motion concept* > *non-concept*. Additionally, search volume correction was used with masks defined by *color* > *baseline* (*p* < 0.001), and *motion* > *baseline* (*p* < 0.001). The baseline condition consisted of null trials in which no stimulus was presented and no task performed.

### Retinotopic mapping procedure

Although others have encountered difficulties in mapping areas beyond V1–V3 retinotopically (Winawer et al., 2010; Wandell and Winawer, 2011), we nevertheless wanted to learn whether concept related activity was associated with higher retinotopically organized areas, especially those within the parietal cortex (Swisher et al., 2007; Silver and Kastner, 2009). Four male participants between the ages of 20 and 40 who had taken part in the main imaging experiment were also scanned for the retinotopic mapping of V4, V5 and areas in the parietal cortex. Polar maps of these areas were calculated using phase-encoded retinotopic mapping techniques (Sereno et al., 1995) and retinotopic visual areas were delineated manually. Stimuli used for the retinotopic scans consisted of a wedge pattern (radius = 8°) rotating smoothly in either a clockwise or anticlockwise direction, around a small fixation cross. Eight cycles were completed for each direction, at a speed of 61.2 s/cycle. To maintain attention toward the retinotopic mapping stimuli, a probe (small gray dot) was briefly flashed (200 ms) at random intervals, and participants asked to report the presence of the probe as quickly as possible, via a button press. The stimulus was a colored version of a publicly available retinotopic mapping stimulus (http://www.fil.ion.ucl.ac.uk/~sschwarz/retinotopy.html), and was displayed using MatLab and Psychtoolbox-3 (http://psychtoolbox.org/HomePage).

### Analysis of retinotopic mapping

For each subject, functional data from the mapping experiments were transformed to phase space, averaged across directions, and overlaid onto the inflated anatomical brain image, generated using FreeSurfer (http://surfer.nmr.mgh.harvard.edu/). The boundaries of the IPS areas, V1, V4, and V5 were delineated by identifying the representation of the vertical and horizontal meridians from the mirror reversals in the phase map. Mask images for these areas were made from these boundaries and average intensities from the concept experiment were evaluated using SPM-8. Retinotopic maps of V1, V4, V5, and IPS areas were calculated for four subjects. Analysis of concept selectivity across IPS areas was performed for all four subjects separately using their individual retinotopic maps. Analysis of concept selectivity within V4 and V5 was also calculated using the normalized and averaged retinotopic maps of the same four subjects. Although this method of normalization leads to a loss of precision, our intention was to define in approximate terms the locations of the V4 and V5, enabling use of these retinotopically defined masks on the normalized group data (*n* = 16). Three subjects were excluded because of poor alignment between functional data and mask images, leading to a total of thirteen subjects for the retinotopic analysis of V4 and V5.

### Eye tracking control experiment

Ten participants ranging in age from 20 to 40, all with normal or corrected to normal vision, took part in an additional eye tracking control experiment, identical in all respect to the fMRI experiment, other than the removal of null (blank) trials. After undergoing the training phase participants completed 150 trials of each stimulus type (color and motion) on the main DMC task. Eye movements (fixation locations, blinks, and pupil dilations) were recorded using the EyeLink 1000 system (SR Research), with a sampling rate of 1000 Hz. Participants rested their chin and forehead on the chin/head support, with ~ 57 cm viewing distance.

## Results

### Behavioral results

**Figure 3:**
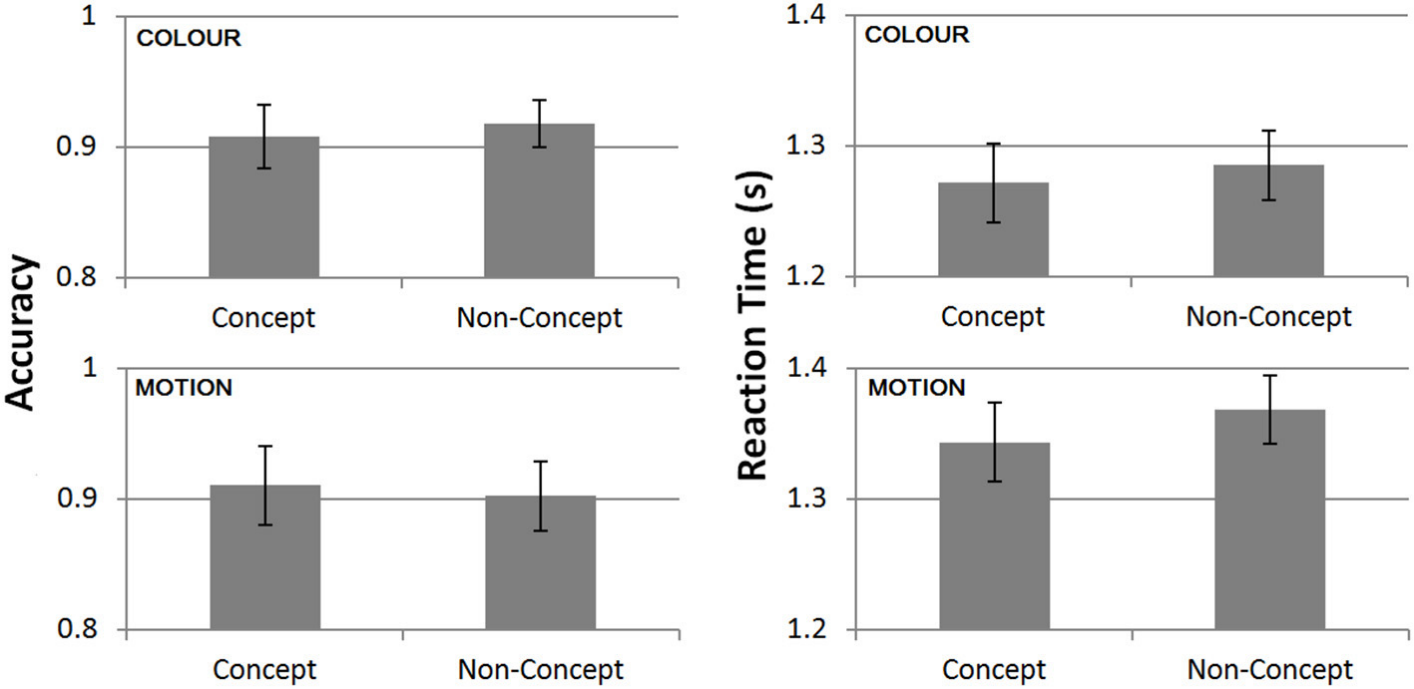
**Task performance from the scanning session averaged across all subjects (*n* = 16)**. Mean accuracies (left) and reaction times (right) split between concept and non-concept conditions. Reaction times (RTs) are plotted relative to the onset of the second stimulus.

We performed paired *t*-tests comparing performance on concept and non-concept trials (Figure 3). No significant differences were found in terms of accuracies [*t*_(15)_ = 0.72, *p* = 0.48] or RTs [*t*_(15)_ = 0.26, *p* = 0.8]. This comparison remained non-significant after exclusion of a single outlier showing shorter concept related RTs [*t*_(14)_ = 0.98, *p* = 0.35]. The similarity in behavioral performance across conditions of interest implies that task difficulty was approximately matched.

### Neuroimaging results

**Figure 4:**
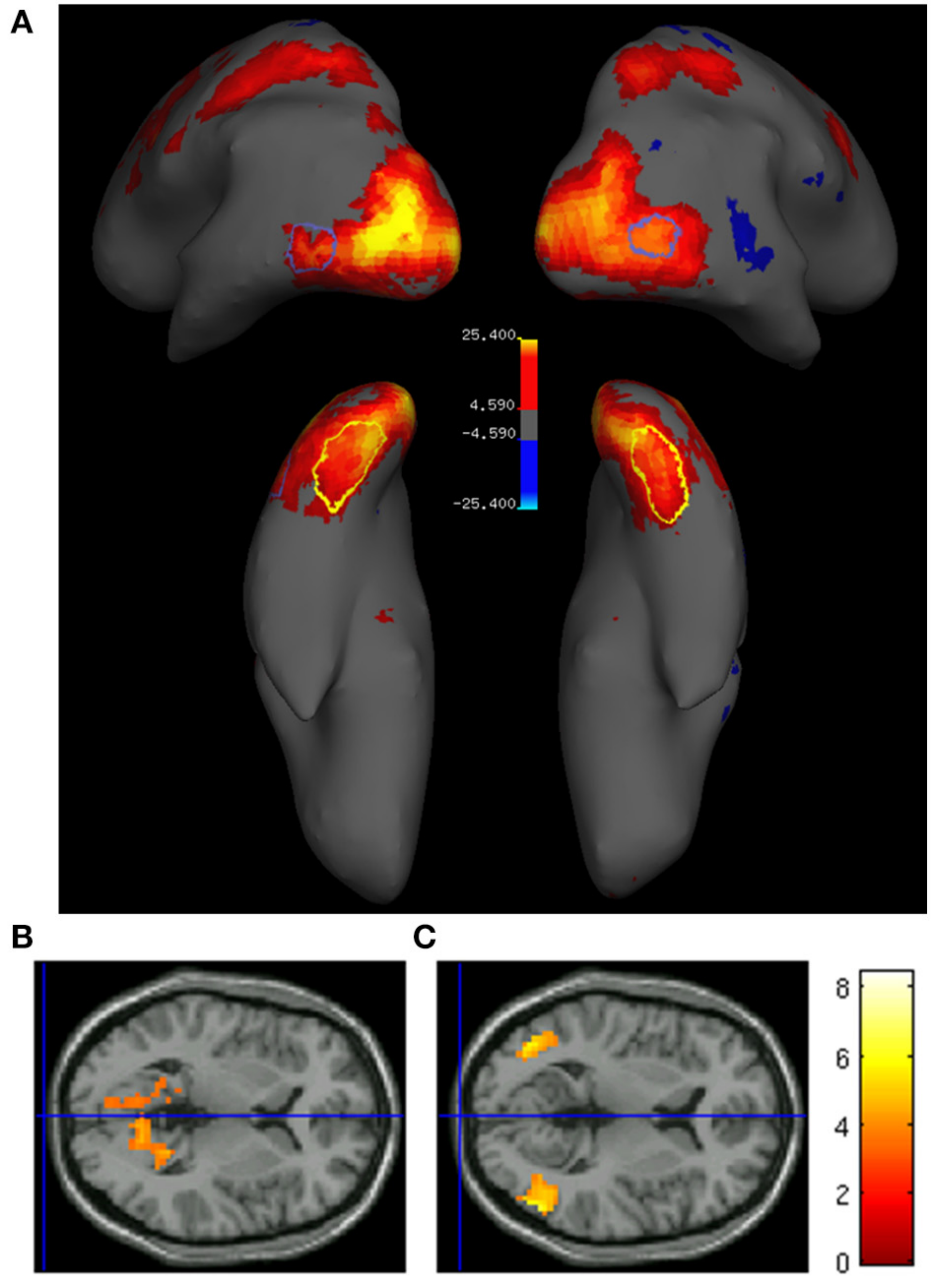
**(A)** Task associated activity (task > baseline; *p* < 0.05 FWE) in a single subject superimposed onto the inflated brain. Yellow/red areas indicate a significantly higher activation for task trials, while blue areas indicate a significantly higher activation for baseline trials. Closed yellow and blue contours indicate the locations of retinotopically mapped V4 and V5, respectively. Bottom row: Transverse sections of the cortex showing group level activity (*n* = 16) superimposed onto the canonical brain, for **(B)** color > motion (MNI *Z* = 3, and **(C)** motion > color (MNI *Z* = 6; *p* < 0.001).

We first compared the BOLD response to color and motion trials, using retinotopic maps, as well as previously published co-ordinates (Table 1), to localize the activity produced by each. The contrast *color* > *motion* produced significant (*p* < 0.05 FWE, cluster level) bilateral activity in a ventromedial region of the occipital lobe (Figure 4B), consistent with the location of the V4 complex (V4 and V4α; Bartels and Zeki, 2000), while the contrast *motion* > *color* produced significant bilateral activation in more lateral and superior areas (Figure 4C), which retinotopic mapping showed to be in V5, an area critical in processing motion signals (Zeki et al., 1991; Braddick et al., 2001; Wandell et al., 2005; Moutoussis and Zeki, 2008; Brouwer and Heeger, 2009, *inter alia*) As expected, activity within retinotopically defined V5 was significantly higher for *motion* stimuli relative to *color* [*t*_(12)_ = 4.1, *p* < 0.01]. Although we found activity in the V4 complex in the contrast *color > motion*, the subdivisions of the V4 complex, and especially its anterior part for which group data showed the strongest activity, were difficult to define retinotopically, possibly because of the same problems encountered by others (Winawer et al., 2010). From our group level results, we are however able to say that the activity was within the V4 complex. The comparison of task trials (regardless of whether they were color or motion) to baseline (no stimuli presented and no task performed) gave strong activation in early visual areas, and also in posterior parietal cortex (Figure 4A).

**Table 1:** **Coordinates (MNI) used for SVCs in areas V4 and V5, inferior parietal, and inferior and superior frontal**.

**Area**	***x***	***y***	***z***	**Study**
V4 (anterior)	−28	−54	−18	Bartels and Zeki, 2000
	28	−50	−16	
V4 (posterior)	−34	−68	−18	Bartels and Zeki, 2000
	34	−74	−14	
V5	38	−62	8	Zeki et al., 1991
	−38	−74	8	
Inferior parietal	−40	−36	42	Chen and Zeki, 2011
	−30	−66	46	
	44	−40	52	
Superior frontal	24	10	56	Chen and Zeki, 2011
Inferior frontal	50	34	18	

*Bartels and Zeki localized V4 by comparing responses from chromatic to responses from achromatic stimuli. Zeki et al. (1991) localized the area V5 by comparing motion to static responses*.

### Activity in V4 and V5 in relation to concept and non-concept stimuli

**Figure 5:**
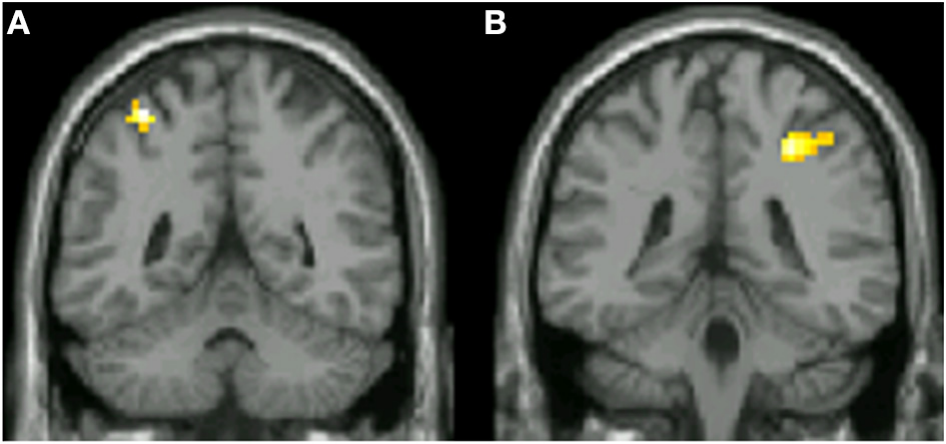
**Coronal slices displaying functional data derived from 16 subjects superimposed onto the canonical brain**. Areas in which significant concept associated activity was found are shown, based on the contrast color concept > color non-concept (*p* < 0.001 uncorrected). Inferior parietal areas are shown in both the left (**A**; MNI *Y* = −50) and right (**B**; MNI *Y* = −37) hemispheres.

Previous studies have shown that areas critical for encoding the shapes of visual objects are also engaged in categorization according to shape (Sigala and Logothetis, 2002; DeGutis and D'Esposito, 2007; Eger et al., 2007). This made it interesting to learn whether early perceptual areas such as V4 and V5 are also involved in concept formation, in this instance according to color and to motion, respectively. No significant difference between *concept* and *non-concept* activity was found in retinotopically defined V4 for color stimuli [*t*_(12)_ = −1.3, *p* > 0.05, n.s.] or retinotopically defined V5 for motion stimuli [*t*_(12)_ = −0.12, *p* > 0.05, n.s]. On average both V4 and V5 showed negative concept related values reflecting a non-significant increase for *non-concept* activity (Figure 5). Additionally, small volume corrections (SVC) were applied to visual areas V4 and V5 bilaterally, localized using previously published coordinates (Table 1, Zeki et al., 1991; Bartels and Zeki, 2000). For all SVCs (reported at *p* < 0.05 FWE, and using a 20 mm radius) there was no significant activity for the contrast *concept > non-concept*. In conclusion, no concept based activity was found in either V4 or V5, based on univariate BOLD signal changes.

### Concept associated activity within the parietal cortex

Our principal aim, however, was to learn whether parietal cortex, and especially different subdivisions within it exhibit concept related activity. In monkeys, V4 and V5 project to an area homologous to human IPS (Seltzer and Pandya, 1980; Rockland and Pandya, 1981; Andersen et al., 1990) terminating in adjacent, juxtaposed regions (Shipp and Zeki, 1995) and this region of parietal cortex has been found to be strongly active in studies of grouping (Zeki and Stutters, 2013). The IPS has been linked to category selectivity in monkey (Freedman and Assad, 2006; Fitzgerald et al., 2011) while surrounding areas of the human inferior parietal cortex have been linked to concept formation (Chen and Zeki, 2011). To address the hypothesis that parietal areas are involved in concept formation related to color or motion, and that distinct sub-regions are engaged in the formation of concepts related to the two attributes, we restricted our search with small volume correction using parietal coordinates previously reported in a concept recognition task (Chen and Zeki, 2011). Contrasting *concept > non-concept*, we found significant (*p* < 0.05, FWE; 20 mm radius) activity bilaterally within the parietal cortex (Figure 5) for color based concepts, and at similar left hemisphere coordinates for motion based concepts. Peak voxel coordinates for all voxels showing significant group level concept related activity are given in Table 2.

**Table 2:** **Peak voxel coordinates given in MNI space, significance (T), and cluster size (kE) of all areas showing concept associated activity in posterior parietal cortex**.

**Feature**	***x***	***y***	***z***	***T***	***kE***
Color	−33	−48	60	6.7	25
	30	−39	45	6.2	66
Motion	−36	−60	60	5.0	5

### Conjunction analysis

**Figure 6:**
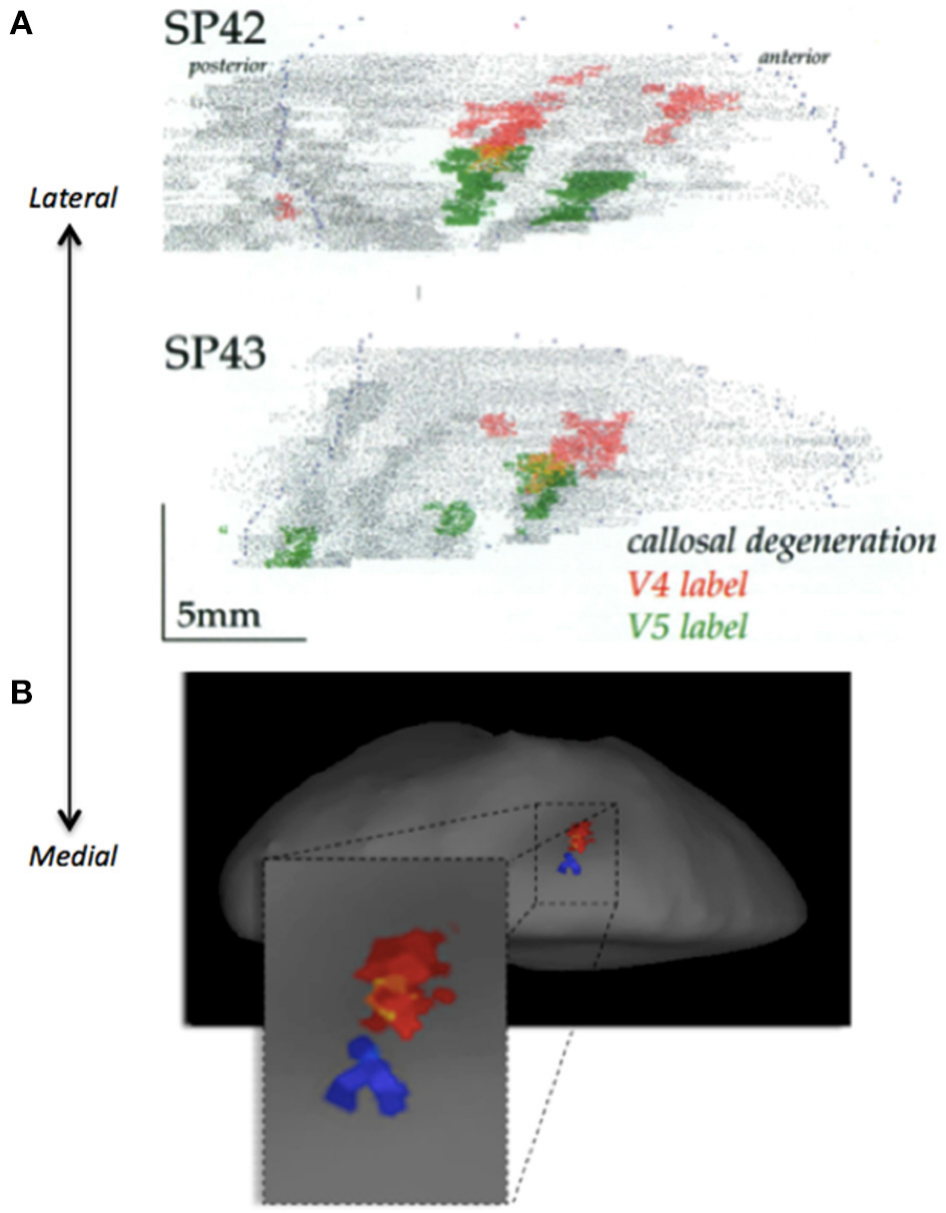
**(A)** Examples of projections from V4 (red) and V5 (green) to the lateral intraparietal sulcus (LIP) in two different monkeys (SP42 and SP43; Reproduced with permission from Shipp and Zeki, 1995). **(B)** Illustration of juxtaposed activity relating to color (red) and motion (blue) concepts. The data is taken from the average of all 16 subjects and superimposed onto the inflated group average structural. Projections from V5 in monkey, and motion related activity in humans, both appear to be more medial, relative to V4 projections in monkey and color activity in humans, appearing more laterally.

To determine whether parietal activity related to color- and motion-based concept signals engage the same or contiguous regions of parietal cortex, we carried out a variant of the conjunction analysis. Because conventional conjunction analysis could not be used due to the shared baseline between conditions (Friston et al., 1999), we generated a mask image containing all voxels significantly more active for concepts compared to non-concepts, collapsed across stimulus type (color and motion). After exclusive masking using this image, parietal activity related to both color or motion concepts remained unchanged. Statistical comparison of concept vs. non-concept activity yielded identical results with or without masking (concept color: *p* < 0.05, FWE bilateral; concept motion *p* < 0.05, FWE right hemisphere). The distinctness of color and motion representations is illustrated in **Figure 8**, showing separable groupings of concept related activity (plotted at a significant level of *p* < 0.001). Consistent with previous anatomical data from monkey (Shipp and Zeki, 1995), the parietal region associated with color appears distinct from, and lateral to, that associated with motion (Figure 6). The result is also consistent with grouping activity based on color and motion (Zeki and Stutters, 2013).

Past evidence also links neurons in the prefrontal cortex (PFC) to category selectivity in monkey (Freedman et al., 2001, 2002, 2003), in addition to the involvement of frontal regions in concept recognition in humans (Chen and Zeki, 2011). We therefore carried out additional SVC analyses within the frontal cortex, centered on previously published coordinates (see Table 1) for the contrasts *color concepts > non-concepts and motion concepts > non-concepts*) yielding no significant concept related increases.

The posterior parietal concept related responses are close to later stage areas of the IPS (areas 3 and 4). Small volume corrections based on the location of IPS 4 (Swisher et al., 2007) yielded significant concept activation (*concept > non-concept*; *p* < 0.05, FWE; 20 mm radius) for both color (bilateral) and motion (unilateral) stimuli. However, the activations do not coincide directly with previously published IPS 4 coordinates (Swisher et al., 2007), appearing to be more dorsal and superior. In order to clarify the relationship between concept activity and individual IPS areas we carried out further analyses.

### Activity related to subdivisions of the parietal cortex

**Figure 7:**
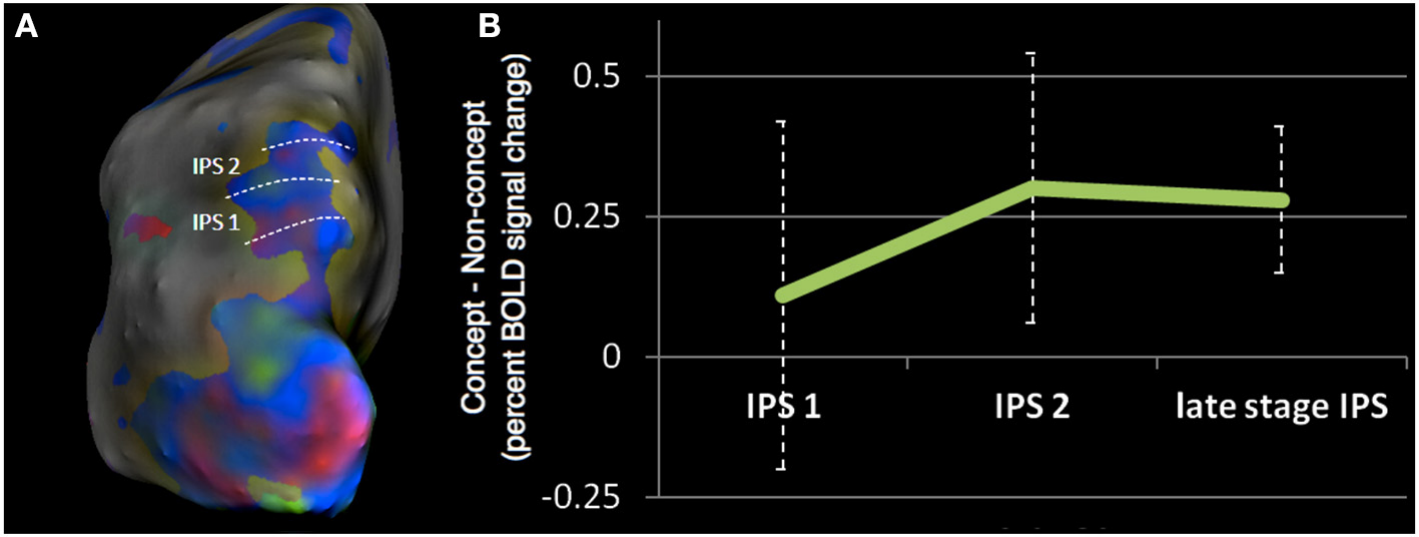
**(A)** Example of retinotopically defined intraparietal sulcus (IPS) areas from a single subject, in which phase data has been projected onto the inflated cortical surface. Color indicates the visual field location of the stimulus associated with peak neural response. Green is associated with stimuli presented in the upper quadrant, blue with stimuli close to the horizontal meridian, and red with stimuli presented in lower quadrant, all presented within the right visual hemifield. **(B)** Mean concept associated activity in retinoptically defined IPS areas, averaged across four subjects. The graph plots the difference between beta coefficients for the concept and non-concept conditions (i.e., the subtraction concept—non-concept). Positive values indicate a greater response to concept stimuli.

Although localizing areas of the parietal cortex is generally a good deal more difficult than the retinotopic definitions of areas V1–V3, a number of distinct retinotopically defined areas have been reported within the IPS (Silver et al., 2005; Swisher et al., 2007). To localize the activity obtained in our study in relation to these subdivisions, we used the results of our retinotopic maps in four subjects. For these, we used a similar mapping stimulus to that of Swisher et al. (2007) who reported reliable delineation of IPS boarders. Using our retinotopic maps we were able to identify the borders between early IPS areas (1–3; Figure 7A). The degree of concept associated activity in IPS 1 and 2, as well as late stage IPS, broadly encompassing IPS areas 3–4, was calculated via the subtraction of *non-concept* from *concept* beta coefficients for each subject. The resultant values were than averaged across all voxels within a single retinotopically defined IPS area. This analysis was performed separately for all subjects with retinotopic maps (*n* = 4). The results revealed highly variable activity in the lower IPS areas (beta differences mean and standard errors, IPS 1: 0.11 ± 0.31, IPS 2: 0.30 ± 0.24), whereas less variable and more robust concept associated activity was observed in late stage IPS regions (0.28 ± 0.13; Figure 7B).

Early subdivisions of the IPS (areas 1–2) have been associated with attentional processing (Silver et al., 2005) since shifting covert spatial attention produces increased activation in IPS 1 and 2. Additionally, the correlation between saccade frequency and BOLD amplitude is strongest in early IPS subdivisions (Konen and Kastner, 2008). Our failure to observe significant concept associated activity in these early areas of the IPS argues against an explanation of the concept related signal in terms of attention, or increased saccade frequency for concept stimuli due to enhanced recognition of target items. We only observed significant IPS activity in late stage areas, beyond areas 1 and 2, consistent with past work reporting IPS category signals even under conditions of equalized attentional demands (Vogels et al., 2002).

### Eye movement control experiment

**Figure 8:**
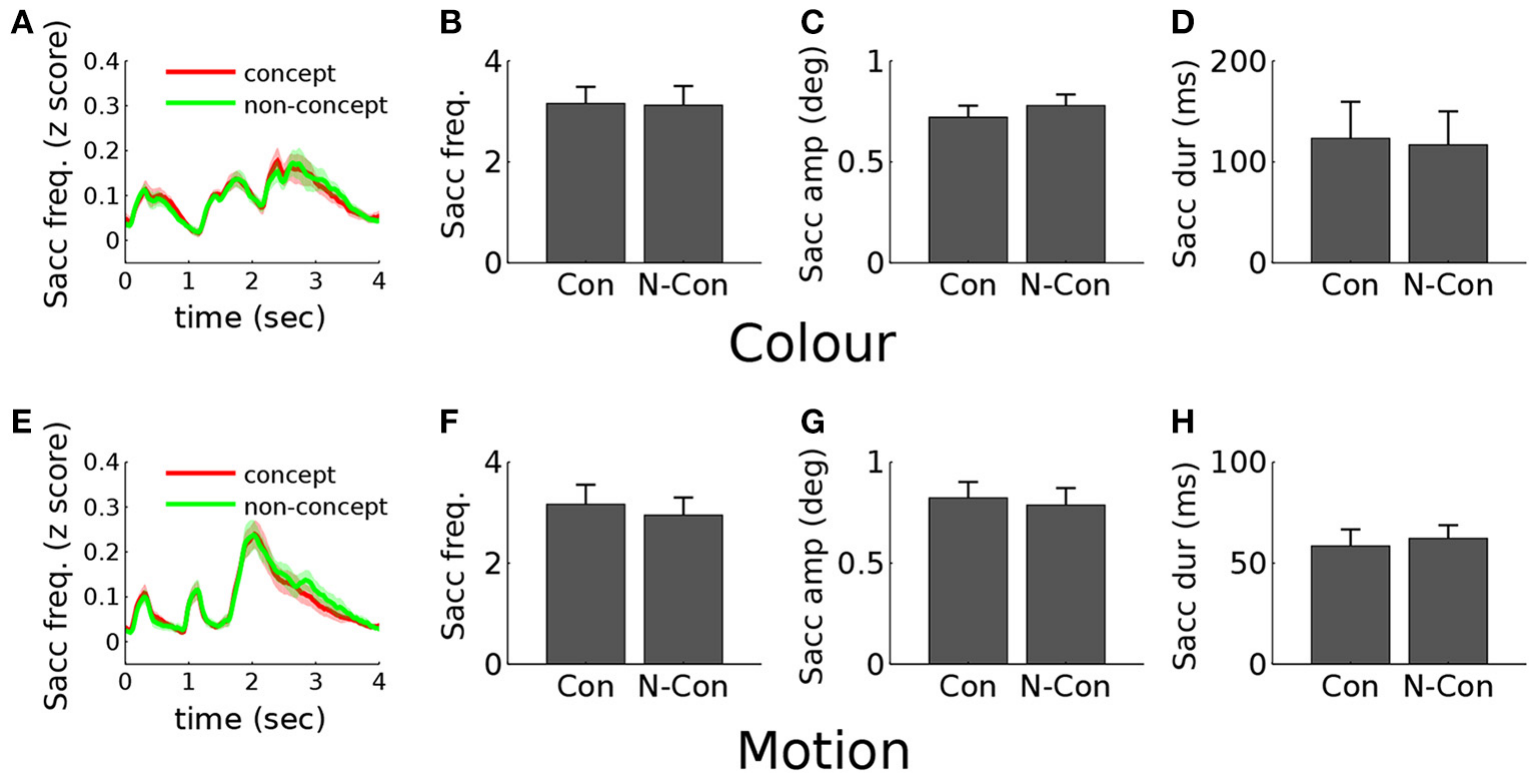
**Eye movement results. (A)** Concept (red) and non-concept (green) saccade frequency profiles for color stimuli plotted from 0 (trial onset) to 4 s post-onset. Stimuli were displayed within the first 2.1 s, and responses (button presses) made within the following period (2–4 s post-onset). **(B)** Color trial mean saccade frequency for concept (Con) and non-Concept (N-Con) conditions, averaged over the stimulus presentation window (0–2.1 s). **(C)** Color trial mean saccade amplitude. **(D)** Color trial mean saccade duration. **(E–H)** Equivalent plots for motion stimulus trials.

To investigate the relationship between the observed posterior parietal activity and eye movement modulations, we carried out an additional eye tracking control experiment on 10 subjects, performing an identical DMC concept formation task to that employed in the main fMRI experiment. Statistical comparison of all metrics (based on both the trial averages) revealed no significant modulation of eye movements across conditions for color stimuli based on the primary eye movement metrics of saccade frequency [*t*_(9)_ = 0.35, *p* = 0.74] saccade amplitude [*t*_(9)_ = 1.4, *p* = 0.20] and saccade duration [*t*_(9)_ = 1.0, *p* = 0.32]. For motion stimuli concept trials were associated with a significantly higher frequency of saccades [*t*_(9)_ = 2.9, *p* ≤ 0.05] but no difference in saccade amplitude [*t*_(9)_ = 0.76, *p* = 0.47] or duration [*t*_(9)_ = 0.67, *p* = 0.52]. Additional statistical comparison of blink frequency and pupil dilation revealed no relationship with concept condition. Figure 8 displays the time-course profiles and trial average results for all saccade metrics. Due to the stimulus specific nature of the eye movement modulations (motion stimuli only) these results fail to provide an explanation for the observed posterior parietal activity. This motion specificity is inconsistent with the fMRI results which show posterior parietal activity for both feature types, with concept related activity being stronger for color than motion stimuli; color is associated with bi-lateral concept activity, and motion only with unilateral. Additionally a purely saccade related modulation fails to explain the juxtaposed and non-overlapping nature of the color and motion parietal activity, instead predicting that activity associated with different feature types should emerge from a common cortical area.

## Discussion

Our principal aim in this work was to study the neural correlates of abstraction, and more specifically to learn whether the functional specialization for grouping visual stimuli according to color and motion that is evident in parietal cortex also applies to the formation of concepts based on these two attributes, with the secondary aim of learning whether areas V4 and V5, which feed the parietal cortex with color and motion signals, have functions related to concept formation, extending their known perceptual roles (Zeki and Stutters, 2013). We chose to concentrate on color and motion because they are more readily separable psychophysically and perceptually than other visual attributes (Ramachandran and Gregory, 1978; Cavanagh et al., 1984; Moutoussis and Zeki, 1997; Cheadle and Zeki, 2011) and because their cortical representations are well separated geographically (Zeki et al., 1991; Watson et al., 1993; Bartels and Zeki, 2000; Wandell et al., 2005; Moutoussis and Zeki, 2008; Murphey et al., 2008; Wade et al., 2008; Brouwer and Heeger, 2009). To do so, we asked participants to learn novel concepts based on these two attributes, and to classify the motion and color stimuli according to whether they could be accommodated within the learned concepts or not. In fact, the concept-related signals we obtained were not from V4 and V5 but from the parietal cortex, to which they both project independently and which is prominently engaged in grouping visual signals according to color or to motion (Zeki and Stutters, 2013).

### Attention

We believe that our results cannot be accounted for by differing attentional demands across conditions because: (a) attentional requirements were equalized across conditions through the use of a delayed match to sample task requiring sustained attention toward multiple stimuli; (b) We did not observe more anterior (e.g., the precentral sulcus or superior frontal sulcus) activity commonly associated with the dorsal fronto-parietal network for top-down control of visual attention (Corbetta and Shulman, 2002; Rust et al., 2006), or heightened activity in perceptual regions (V4/V5) previously associated with attention to color and to motion, respectively (Chawla et al., 1999; Bartels and Zeki, 2005); (c) parietal areas related to concepts derived from different visual features were non-overlapping, making it unlikely that these signals reflect changes in a general attentional network.

### Parietal cortex

Although traditional dissociations between “what” and “where” pathways attribute a distinct spatial role to parietal areas, accumulating evidence links the same parietal areas to a range of functions in humans (Altmann et al., 2005; Schendan and Stern, 2008; Silver and Kastner, 2009; Wei et al., 2011) which cannot be easily accommodated within the “what” and “where” distinction. IPS activity has been linked to a number of functions including binding of different visual features for IPS 4/5 (Wei et al., 2011), mental rotation, working memory and categorization (Schendan and Stern, 2008). More significantly for the present results, parietal activity appears to be of central importance for the formation and maintenance of concepts (Freedman and Assad, 2006; Chen and Zeki, 2011; Fitzgerald et al., 2011), as well as the grouping of visual signals (Zeki and Stutters, 2013). Our results complement and extend these to show a functional specialization within the territory of parietal cortex during the elaboration of concepts based on color and motion, and that color and motion related activity there is juxtaposed, just as in the monkey the projections from V4 and V5 to the parietal cortex are juxtaposed but not overlapping (Shipp and Zeki, 1995). This also extends the cortical specialization for color and motion beyond a perceptual level, to that of concept formation. The results of Braddick et al. (2001) show that form and motion coherence also lead to activity in distinct parts of the intraparietal sulcus, thus strengthening the evidence in favor of a functional specialization for both grouping and concept formation within parietal cortex. The posterior parietal cortex has also been proposed to play an important role in numerosity (Hubbard et al., 2005; Piazza and Izard, 2009), but it should be noted that although the task used in the present study required subjects to count the number of occurrences of stimulus types, these demands were equal across conditions.

The territory of parietal cortex involved includes posterior parietal cortex and the IPS. In combination, these results support the hypothesis that these parietal stages form part of the nexus between perception and cognition, where perceptual information undergoes a transformation into the cognitive domain (Freedman and Assad, 2011). Although episodic memory formation has been reported to engage posterior parietal cortex (see Uncapher and Wagner, 2009, for a review) our conclusion is that representations in these parietal areas are not limited to specific instances (or episodes) but encompass knowledge that generalizes across instances (i.e., categorical and conceptual knowledge). The direct anatomical connections between parietal regions and PFC (Ungerleider et al., 1989; Webster et al., 1994) enable the passing of category-based information between the two areas, consistent with reports of category selectivity within the PFC (Freedman et al., 2001, 2002, 2003). Although category based signals exhibit shorter latencies in parietal areas, compared to the PFC (Swaminathan and Freedman, 2012) indicative of a primary role for the parietal cortex, our result do not exclude the possibility that category signals are first computed elsewhere (e.g., the PFC) and are subsequently fed back to modulate activity in parietal color or motion selective areas.

### The role of earlier visual areas V4 and V5

The color- and motion- based visual inputs to IPS come from the visual areas of prestriate cortex and it seemed plausible that they, too, should show some activity related to the formation and maintenance of concepts. Indeed, previous studies have shown that areas involved in processing shapes are also involved in their categorization (Sigala and Logothetis, 2002; DeGutis and D'Esposito, 2007; Eger et al., 2007). We observed no concept related increase in the average BOLD signal strength in either V4 or V5. A possible explanation for this is that, within a single cortical area, learning may simultaneously enhance and suppress responses of separate neural populations, compatible with theories of perceptual or category learning based on sharpening of the neural tuning curve (Hoffman and Logothetis, 2009). On the other hand, it may simply reflect the fact that while these areas are involved in an important stage of concept formation, namely grouping, they are not further directly involved in the more abstract process of concept formation.

### Conclusion

In summary, we have presented evidence for the involvement of parietal areas in the generation of concepts based on color and motion. The IPS is in an ideal position to use the information conveyed by earlier level visual regions in forming more abstract concepts or categories (Fitzgerald et al., 2011; Freedman and Assad, 2011). Our results support the hypothesis that parietal areas are important for the formation and maintenance of concept knowledge.

### Conflict of interest statement

The authors declare that the research was conducted in the absence of any commercial or financial relationships that could be construed as a potential conflict of interest.
